# Supplementary material for: Tumorigenicity of EGFR- and/or HER2-Positive Breast Cancers Is Mediated by Recruitment of Tumor-Associated Macrophages
Source: Int J Mol Sci. 2023 Jan 11;24(2):1443. doi: 10.3390/ijms24021443 (PMC9866454; doi:10.3390/ijms24021443)
Supplement: Supplementary file 1 [file ijms-24-01443-s001.zip › Supplementary Figure Legends.pdf]

Supplementary Figure Legends

Supplementary Figure S1. HER2 overexpression leads to increase of CCL2 transcript levels in Hs578T cells. Transcript levels of *HER2* and *CCL2, in* Vec and HER2-overexpressed Hs578T cells were analyzed by real-time PCR. Values were normalized to *ACTB.* *, $P < 0.05$

Supplementary Figure S2. Effect of conditioned culture media of HER2 overexpressing cells on the expression of *CCL2*, *CCL5*, and *IL-6*. (A, B, C) Transcript levels of *CCL2, CCL5,* and *IL-6* following treatment with conditioned culture media obtained from Vec and HER2-overexpressed MDA-MB231 cells were analyzed by real-time PCR. Values were normalized to *ACTB. ns,* non-significant.

Supplementary Figure S3. Effect of Trastuzumab on the expression of *CCL2*, *CCL5*, *IL-6*, and *IL-8*. (A, B, C, D) Transcript levels of *CCL2, CCL5, IL-6, and IL-8* in Vec and HER2-overexpressed MDA-MB231 cells treated with 50 μM trastuzumab were analyzed by real-time PCR. Values were normalized to *ACTB. ns,* non-significant; *, $P < 0.05$; **, $P < 0.005$.

Supplementary Figure S4. Effect of neratinib on the expression of *CCL5*, *IL-6*, and *IL-8*. Transcript levels of *CCL5, IL-6, and IL-8* in Vec and HER2-overexpressed MDA-MB231 cells treated with 2 μM neratinib were analyzed by real-time PCR. Values were normalized to *ACTB.* *, $P < 0.05$; ϕ, $p < 0.05$.
