# Supplementary material for: Tumorigenicity of EGFR- and/or HER2-Positive Breast Cancers Is Mediated by Recruitment of Tumor-Associated Macrophages
Source: Int J Mol Sci. 2023 Jan 11;24(2):1443. doi: 10.3390/ijms24021443 (PMC9866454; doi:10.3390/ijms24021443)
Supplement: Supplementary file 1 [file ijms-24-01443-s001.zip › Supplementary Materials and Methods.pdf]

Supplementary Materials and Methods

Generation of stable HER2-overexpressed Hs578T cells

Hs578T cells were seeded in 6-well plates. Cells were transfected with either empty vector or constitutively active HER2 vector (Addgene, Cambridge, MA, USA). Effectene (Qiagen) was used according to the manufacturer's protocol. Cells were maintained in culture media with Effectene for 48 hours. For stable transfection, cells were selected with G418.
