# Supplementary material for: Tumorigenicity of EGFR- and/or HER2-Positive Breast Cancers Is Mediated by Recruitment of Tumor-Associated Macrophages
Source: Int J Mol Sci. 2023 Jan 11;24(2):1443. doi: 10.3390/ijms24021443 (PMC9866454; doi:10.3390/ijms24021443)
Supplement: Supplementary file 1 [file ijms-24-01443-s001.zip › Supplementary Table S1.pdf]

Supplementary Table S1. Primer sequences used for real time-polymerase chain reactions

Gene name	Primer	Sequence (5’ to 3’)
ACTB	Forward (F)	TCACCATTGGCAATGAGCGGTT
	Reverse (R)	AGTTTCGTGGATGCCACAGGACT
EGFR	F	CATGTCGATCTTCCAGA
	R	GGGACAGCTTGGATCACACT
HER2	F	CACTTCAACCACAGTGGCAT
	R	ATTCACATACTCCCTGGGGA
CCL2	F	CAGCCAGATGCAATCAATGC
	R	GCACTGAGATCTTCCTATTGGTGAA
CCL5	F	CCTCGCTGTCATCCTCATT
	R	AGCAGCAGGGTGTGGTGTC
IL6	F	AATTCGGTACATCCTCGACGG
	R	GGTTGTTTTCTGCCAGTGCC
IL8	F	AGGGTTGCCAGATGCAATAC
	R	AAACCAAGGCACAGTGGAAC
IL1B	F	GCCCTAAACAGATGAAGTGCTC
	R	GAACCAGCATCTTCCTCAG
CD11B	F	CAGCCTTTGACCTTATGTCATGG
	R	CCTGTGCTGTAGTCGCACT
