# Supplementary figures and images for: Tumorigenicity of EGFR- and/or HER2-Positive Breast Cancers Is Mediated by Recruitment of Tumor-Associated Macrophages
Source: Int J Mol Sci. 2023 Jan 11;24(2):1443. doi: 10.3390/ijms24021443 (PMC9866454; doi:10.3390/ijms24021443)

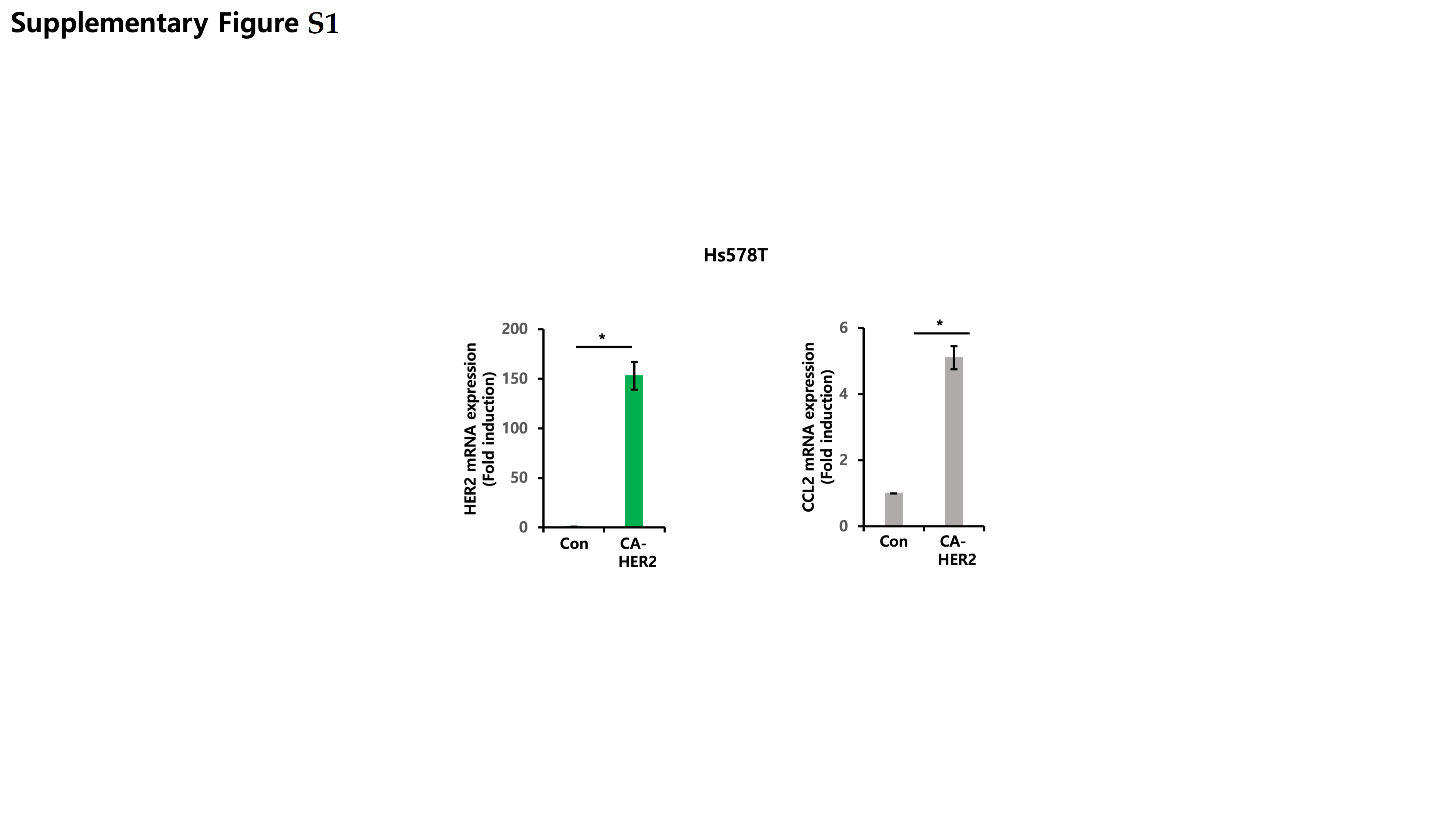

Supplement: Supplementary file 1 [file ijms-24-01443-s001.zip › FigS1.tiff]

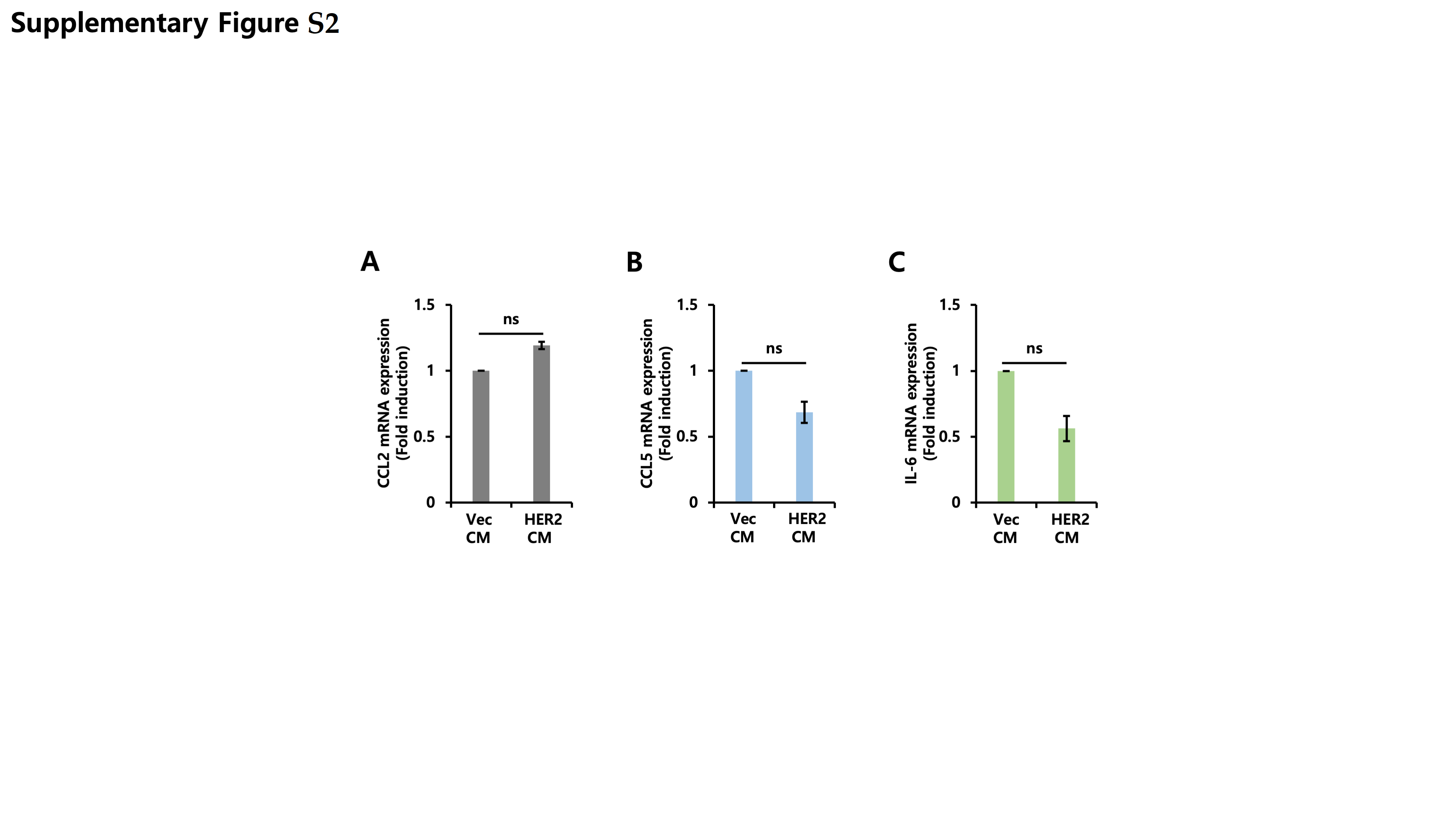

Supplement: Supplementary file 1 [file ijms-24-01443-s001.zip › FigS2.tiff]

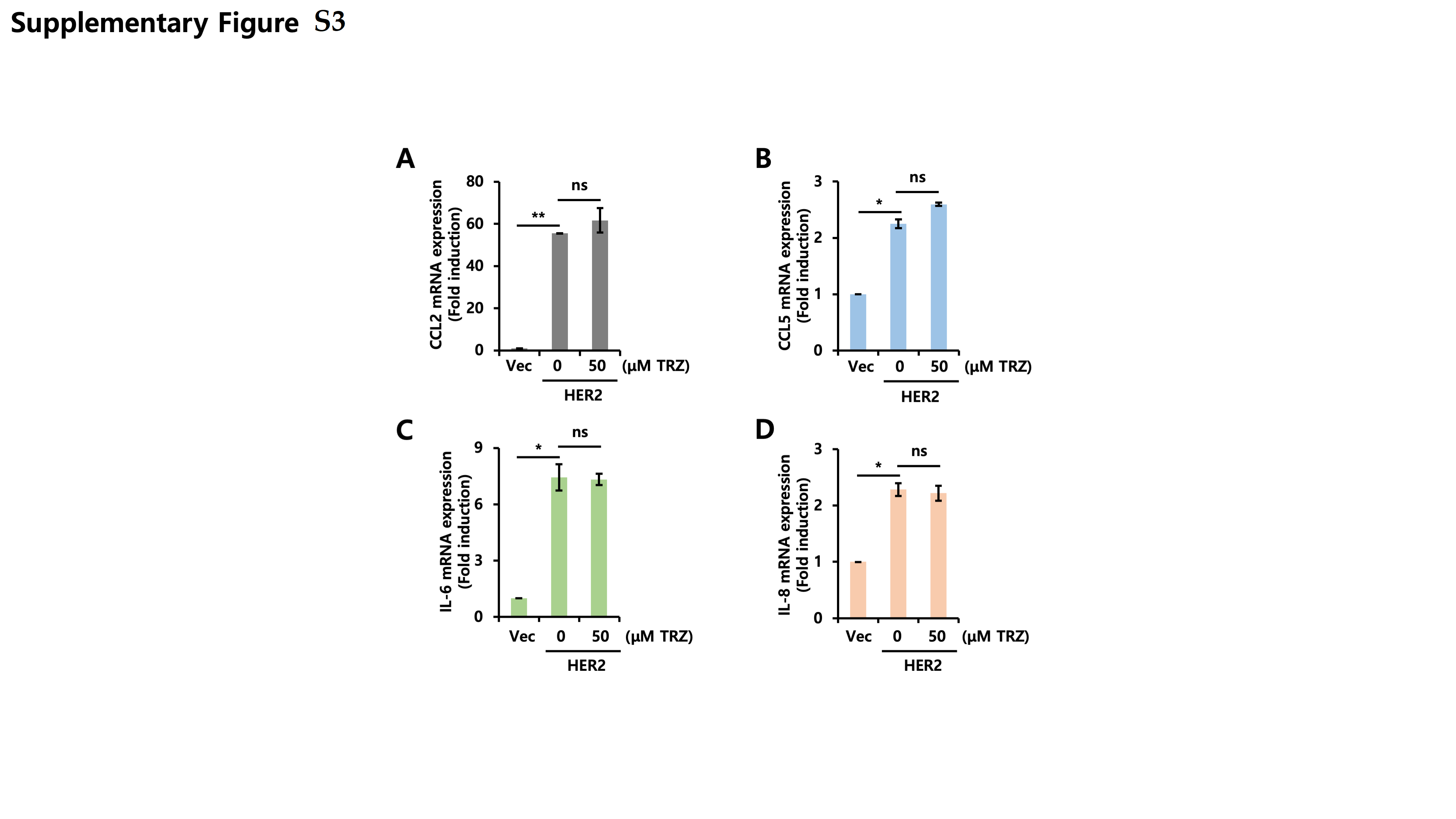

Supplement: Supplementary file 1 [file ijms-24-01443-s001.zip › FigS3.tiff]

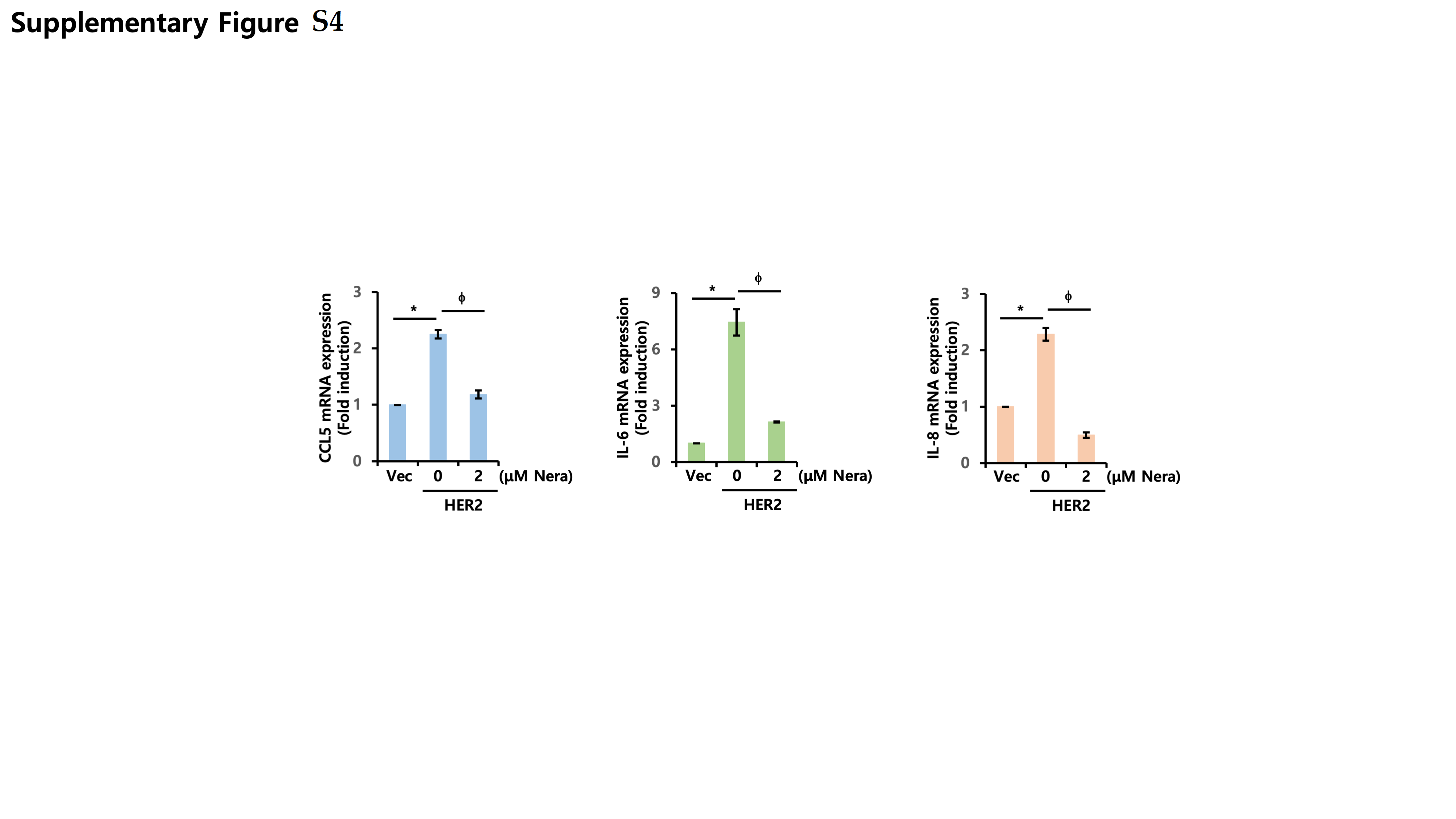

Supplement: Supplementary file 1 [file ijms-24-01443-s001.zip › FigS4.tiff]
